# The Conclusive and Continuous Tool to Assess Severity and Improvement of Eating Disorders (CONTASI-ED): Development and Psychometric Properties

**DOI:** 10.3390/nu17111790

**Published:** 2025-05-24

**Authors:** Moria Golan, Roni Sides, Keren Baum, Rachel Arbib, Wiessam Abu Ahmad

**Affiliations:** 1 Department of Nutritional Sciences, Tel Hai College, North Galilee, Qiryat Shemona 1220800, Israel; 2 SHAHAF—A Community-Based Facility for Treatment of Eating Disorders, Ganey Hadar 7683000, Israelrachelarbib@gmail.com (R.A.); 3 Braun School of Public Health, Hebrew University-Hadassah, Jerusalem 91120, Israel; wiessam.abuahmad@mail.huji.ac.il

**Keywords:** eating disorders, assessment, severity, longitudinal, psychometric properties

## Abstract

**Background:** Accurately assessing eating disorder (ED) severity and treatment progress is essential for effective intervention. The Comprehensive and Continuous Tool to Assess Severity and Improvement of Eating Disorders (CONTASI-ED) was developed to address limitations in existing assessments by incorporating behavioral, cognitive, and physiological markers. **Objectives:** This study aimed to examine the psychometric properties and sensitivity to symptom changes of the CONTASI-ED in a community-based clinical sample of women with ED. **Methods:** Participants were 58 females diagnosed with EDs and 10 healthy controls. The CONTASI-ED assessments were conducted over multiple time points in outpatient and intensive treatment settings. We examined reliability, validity, and sensitivity to treatment-related change. The CONTASI-ED scores were compared with EAT-26, and multivariable analyses explored the effects of body mass index (BMI), age, and post-traumatic stress disorder (PTSD) on symptom trajectories. **Results:** The CONTASI-ED demonstrated strong reliability, with test–retest correlations between 0.72 and 0.90 and inter-rater reliability of 0.68–0.95. The tool effectively distinguished ED patients from healthy controls (*p* < 0.001) and correlated strongly with EAT-26. Significant reductions in the CONTASI-ED scores over time (*p* < 0.001) reflected treatment-related improvements—although temporary score increases highlighted greater self-awareness and symptom disclosure. BMI, age, and PTSD significantly influenced symptom severity and treatment response. **Conclusions:** The CONTASI-ED demonstrated strong reliability and validity in distinguishing clinical and non-clinical cases and in tracking treatment-related changes. However, the findings are based on a relatively small, all-female sample, underscoring the need for further validation in more diverse populations.

## 1. Introduction

The clinical assessment of eating disorders (EDs) remains a significant challenge due to their complex, heterogeneous, and often fluctuating symptom presentations. EDs manifest in a wide range of physical, cognitive, and emotional symptoms, varying widely across individuals. Over the past decades, a wide range of validated assessment tools has been developed, each with distinct strengths and clinical applications. Existing screening tools have high specificity and sensitivity, yet many are impractical in primary care due to complexity, cultural adaptability issues, or administration demands [1,2]. Clinicians often rely on a combination of assessment tools to quantify illness severity over time or detect meaningful clinical changes in ED pathology [3,4,5].

Tools such as the Eating Disorder Inventory-3 (EDI-3) [6,7] and the Eating Disorder Examination (EDE) [8,9] have demonstrated diagnostic precision and contributed valuable insight into the cognitive and emotional correlates of EDs. The EDI-3 also provides valuable insight into psychological traits linked to EDs—such as perfectionism and low self-esteem [6,7]. However, it is time-consuming and better suited for diagnostic profiling than for ongoing outcome monitoring.

The Eating Disorder Examination (EDE), a well-validated interview protocol, is similarly robust. Yet, it requires specialized training and lengthy administration [8,9]. The EDE-Q offers a more practical self-report alternative but may be influenced by social desirability or lack of insight, particularly in individuals with poor insight or profound shame [10]. Several other tools focus on symptom tracking or life impact. For instance, the CHEDS [11] measures session-by-session fluctuations but lacks coverage of psychiatric comorbidities. The Munich ED-Quest [12] aligns with DSM-5 criteria but overlooks factors such as depression and trauma, which are crucial for predicting treatment outcomes [13].

Broader tools such as the Eating Disorder Quality of Life (EDQoL) Scale assess quality-of-life impacts but may not detect short-term symptom shifts, and thus are often used as supplementary measures [14]. Similarly, the CR-EAT captures long-term patterns but is less responsive to rapid clinical changes, limiting its utility for real-time decision-making [15].

Newer methods, such as the Ecological Momentary Assessment (EMA), use digital tracking to reduce recall bias, but lack standardization in scoring and interpretation, and are not designed to estimate overall illness severity or track treatment progress [16].

Common screening tools such as the SCOFF questionnaire [17], the EAT-26 [18], and the EDE-Q [19] are often based on cutoff scores and may miss atypical presentations—such as restrictive eating disorder (RED) patients who maintain normal weight despite severe metabolic dysregulation [20], or EDs in males, older adults, and culturally diverse populations [1,21]. For example, individuals from cultures where weight gain is perceived as a sign of health or prosperity may experience EDs differently and focus more on control or ritualistic eating behaviors than on body dissatisfaction [22,23]. These cultural and clinical nuances highlight the need for more inclusive, flexible tools that can account for diverse symptom profiles and trajectories. Moreover, the continued reliance on static indicators like BMI presents another limitation. While BMI is a relevant clinical parameter, it explains less than 15% of the variance in ED-related quality of life [24] and may obscure symptom severity in higher-weight individuals or those with normo-weight presentations.

To summarize, current assessment tools show limited sensitivity to change, insufficient coverage of comorbid factors, and overreliance on static indicators like BMI.

### 1.1. The Need for a Comprehensive and Continuous Assessment Tool

The Comprehensive and Continuous Tool to Assess Severity and Improvement of Eating Disorders (CONTASI-ED) was developed to address these challenges. Rather than replacing existing measures, the CONTASI-ED aims to complement them by integrating behavioral, cognitive, physiological, and psychosocial domains into a brief, structured format suitable for routine clinical use.

Grounded in contemporary evidence [25,26,27], The CONTASI-ED incorporates both state-based indicators (e.g., purging, somatic symptoms) and more stable trait-level contributors (e.g., compulsiveness, trauma history). This enables clinicians to track intra-individual changes over time, detect relapse risk, and support timely treatment adjustments. Its modular, transdiagnostic structure also increases applicability across ED subtypes and care settings.

To our knowledge, the CONTASI-ED is the first tool to combine this breadth of content with a scoring system sensitive enough for short-term progress tracking, while remaining feasible for real-world implementation.

### 1.2. Study Aims

This study aimed to evaluate the psychometric properties of the CONTASI-ED—including reliability, validity, and sensitivity to within-person symptom changes over time. Secondary analyses explored how the CONTASI-ED scores varied by BMI, age, and PTSD diagnosis, and how they compared with EAT-26, an established screening tool.

## 2. Methods

### 2.1. Development of CONTASI-ED

The development of the CONTASI-ED followed a structured, multi-phase process aimed at generating a clinically actionable instrument that captures the complexity of ED symptomatology and its evolution over time.

An initial item pool was generated based on clinical records, established ED instruments, and key domains identified in recent literature. Items were reviewed for redundancy, ambiguity, and contextual relevance, and were then organized into conceptual domains that reflected both theoretical frameworks and clinical utility. A multidisciplinary expert panel—including clinicians from psychiatry, psychology, nutrition, and research—refined the draft tool over two iterative rounds, focusing on clarity, scoring feasibility, and alignment with treatment decision-making. The tool was subsequently pilot tested across three clinical settings (inpatient, day program, and outpatient), leading to minor revisions in wording, item structure, and scoring anchors. The final tool was designed to be brief (completion time < 20 min), adaptable for self-report or clinician administration, and sensitive to both overt symptoms and underlying contributors to ED severity.

### 2.2. Item Generation and Tool Structure

Item content for the CONTASI-ED was informed by established ED assessment models and empirical literature [2,4,11,14,28,29,30,31,32,33,34,35]. The initial version included 78 items across eight candidate domains. During the refinement phase, concept saturation was monitored, and no new themes emerged, supporting content sufficiency. Following expert review, the final version included 61 items organized into six domains: Starting Point, Anthropometrics and Menstrual Cycle, Pathophysiology, Self-Care, Compulsiveness, and Obsessiveness.

The tool is structured as a checklist with standardized scoring anchors and a summed severity score. Item content includes behavioral symptoms (e.g., restriction, purging), cognitive-affective factors (e.g., intrusive thoughts), psychosocial history (e.g., trauma, treatment dropout), and a limited number of physiological markers (e.g., bradycardia, abnormal electrolyte or enzyme levels), which are commonly used in ED medical monitoring. These physiological items were included not as diagnostic thresholds, but as clinically relevant indicators that can support comprehensive severity profiling and facilitate interdisciplinary communication.

Higher total scores reflect greater illness severity and clinical complexity. Full scoring details and domain structure are provided in Table 1.

### 2.3. Expert Panel Review and Scoring

An expert panel consisting of one psychiatrist, two clinical psychologists, four dietitians, and one statistician, all with substantial experience in eating disorder treatment—reviewed the tool for clinical relevance, clarity, and scoring feasibility.

The review process involved two formal consensus rounds. In Round 1, panelists rated each item on a 5-point Likert scale for clarity, clinical relevance, and contribution to illness severity and relapse risk. Items with high variability or low median ratings were flagged for further discussion. In Round 2, a structured Delphi method was employed to resolve disagreements and finalize the scoring schema. Consensus was defined as ≥75% agreement on each item’s inclusion, phrasing, and weight.

Final weights were determined using a dual approach. Exploratory Factor Analysis (EFA) was used to identify item-level loadings, with those above 0.50 prioritized within their respective domains. These empirical results were supplemented with clinical judgment from the expert panel, particularly regarding each item’s relevance to treatment decision-making and relapse prevention [3,4]. For instance, although some physiological items (e.g., bradycardia, CPK levels) showed modest loadings, they were retained at lower weights due to their clinical importance in medical risk assessment. Conversely, items reflecting obsessive thoughts or rigid behavioral patterns received higher weights, reflecting their consistent association with treatment resistance and relapse. Scoring anchors were standardized to improve consistency across raters and treatment contexts.

The tool was designed for flexible administration and can be used either as a clinician-administered interview or as a structured self-report questionnaire, depending on the setting and user preference. This flexibility enhances its usability across a wide range of clinical environments.

The final version of the CONTASI-ED includes six weighted domains, contributing to a total possible score of 101. These domains are outlined in Table 1, which details the number of subcategories, number of items, maximum scores, and examples of the key indicators included in each domain.

### 2.4. Pilot Testing

The study protocol was approved by the institutional review board of Tel Hai Academic College on 3.2021. Informed consent was obtained in writing from participants and their legal guardians.

This pilot study was conducted to evaluate item clarity, response feasibility, and clinical relevance. The preliminary version of the CONTASI-ED was pilot-tested by eight clinical dietitians across three different clinical settings: inpatient unit, intensive day treatment program, and outpatient clinics. During the pilot phase, clinicians completed the proposed tool alongside the EAT-26 questionnaire for all their patients and provided structured feedback through written comments and group debriefing meetings. Based on this feedback, 11 items were revised to enhance clarity, improve the comprehensibility of behavior descriptions, and align more closely with clinical language.

Key modifications included the addition of items related to previous treatment history and occupation; recoding of several categorial variables into continuous scales for greater sensitivity in capturing symptom severity; and inclusion of emotional and regulatory indicators commonly observed in comorbid presentations—such as mood disorders, anxiety, and impulse control disorders, which often underlie eating pathology.

These modifications aimed to improve the comprehensiveness and clinical interpretability of the tool. The revised version was subsequently evaluated for reliability and validity in a naturalistic clinical sample, where severity levels tend to vary continuously and do not align with categorical diagnostic threshold (Supplementary Materials S1).

### 2.5. Statistical Analysis

To evaluate the psychometric properties of the CONTASI-ED and its sensitivity in tracking longitudinal changes in illness severity and treatment outcomes, a series of statistical analyses were performed:

The dataset was reformatted from a wide to a long format to facilitate time-series analysis. Participants with at least two valid BMI measurements were included.

Each time point was modeled within a unified linear mixed-effects model, which accounts for repeated measures and estimates fixed effects jointly across the full-time series. As such, adjustment for multiple comparisons was not required, since the structure of the model controls for overall type I error without inflating significance at individual time points.

Additionally, multivariable models incorporated age and PTSD diagnosis as covariates to control for potential confounding effects. These adjustments improved model fit, as reflected in increased speculative marginal R^2^ values, and enabled a more precise estimation of independent effects.

Although diagnostic categories (e.g., anorexia nervosa, bulimia nervosa, binge eating disorder) were available, analyses were conducted using BMI-based groups. This approach was chosen due to diagnostic fluidity across time points, particularly in patients transitioning between restrictive and binge-purge behaviors. Grouping by BMI allowed a clearer interpretation of symptom trajectories, minimized misclassification, and aligned with the tool’s aim to track severity across a transdiagnostic spectrum. Thus, to investigate how the CONTASI-ED scores varied across different BMI categories, the BMI from the first three valid time points was calculated for each participant. Based on this, participants were categorized into three groups: Underweight, Normal Weight, and Overweight.

All analyses were conducted using R statistical software (version 3.5.0). Longitudinal data were analyzed using linear mixed-effects models, with random intercepts at the participant level, to account for repeated measurements. The model included Time as a fixed effect to assess the trajectory of symptom changes. An interaction term between Time and BMI group was introduced to determine whether the patterns of the CONTASI-ED and EAT-26 scores differed across BMI categories. Univariate and multivariate survival analyses were also performed to explore the predictive value of the CONTASI-ED scores for treatment outcomes. The CONTASI-ED and EAT26 scores were analyzed to examine changes over time and the effect of BMI group status on those changes.

To visualize the longitudinal trends, line plots were generated, displaying the mean CONTASI-ED and EAT-26 scores over time for each BMI group. To better understand the trend of EAT-26 measurements over time across BMI groups, we applied the locally estimated scatterplot smoothing (LOESS) method. Since the CONTASI-ED involved more frequent measurements than EAT-26, gaps in the data related to the latter made it difficult to observe a clear trend using traditional line plots. The LOESS method was used to smooth fluctuations and provide a continuous representation of the trajectory over time. This approach allowed us to visualize the overall trend of EAT26 scores while preserving local variations, ensuring a more accurate interpretation of changes within each BMI group.

### 2.6. Power Calculation

A post hoc power analysis was conducted to assess the adequacy of the sample size. Based on the observed effect sizes from the linear mixed-effects models, ranging from −12.75 to −22.54 units in STAT scores over time, and standard errors between 1.3 and 1.8, we estimated the statistical power with a two-sided alpha of 0.05 and an intra-class correlation (ICC) of 0.8. With 39 participants and 481 repeated observations, the power to detect a mean difference of 12.75 units was approximately 99%, and exceeded 99.9% for larger differences. These results support that the available sample size was sufficient to detect the longitudinal changes in STAT scores with high precision.

## 3. Results

### 3.1. Study Population

All participants were recruited and assessed in Israel. All procedures adhered to the Declaration of Helsinki. Assessments took place within community-based clinics, hospital-affiliated outpatient programs, and intensive day programs. This study included 58 Israeli female patients, who were admitted to community-based outpatient clinics for eating disorders during 2022–2023. Data from repeated assessments of 31 patients over 22 time points, and from the remaining 17 patients (who had not remained in treatment long enough) at 3 time points, were incorporated into the psychometric analysis of the CONTASI-ED. Ten other patients from this sample were treated in an intensive day program, with the CONTASI-ED assessments conducted over three months. There were no statistically significant differences in mean age or years of education among the three participant groups (Table 2). All participants provided written consent to have their data used for this study’s analysis and publication.

### 3.2. Psychometric Properties

The psychometric evaluation of the CONTASI-ED revealed strong reliability and validity across multiple measures, as follows:

Test–retest reliability: Pearson’s correlation coefficients (r) for test–retest reliability of the total CONTASI-ED score ranged from 0.72 to 0.90—indicating excellent stability over time.

Inter-rater reliability: Three clinicians independently interviewed and scored 22 patients. Fleiss’s Kappa reliability (κ) ranged from 0.68 to 0.95—reflecting high agreement between different raters using the tool.

Patient–dietitian reliability: Spearman correlations between patient self-reports and dietitian-administered assessments ranged from 0.75 to 0.92—indicating strong alignment between the two modes of administration.

Criterion (discriminant) validity: The CONTASI-ED demonstrated strong criterion validity.

**Figure 1:**
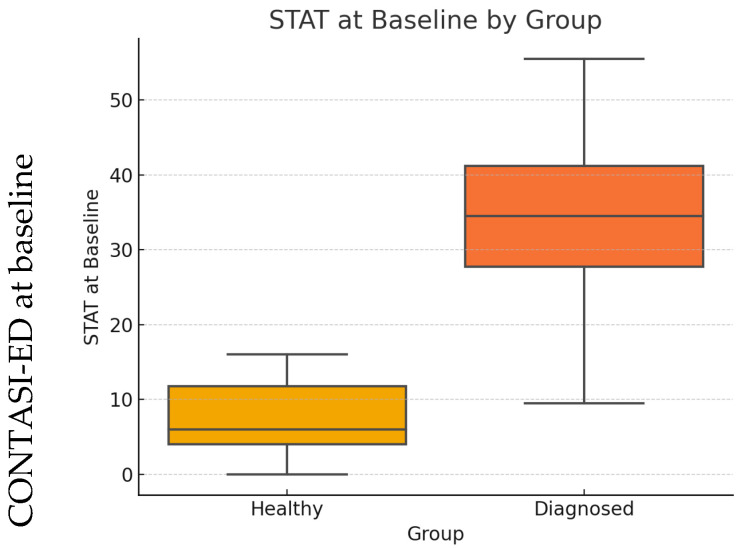
CONTASI-ED at baseline for healthy vs. ED-diagnosed patients.

At baseline, scores were significantly higher among ED patients compared to healthy controls (*p* < 0.001; see Figure 1).

A parallel analysis using the EAT-26 yielded a similar pattern, with both tools showing large effect sizes and clear discrimination between groups, as summarized in Table 3.

**Figure 2:**
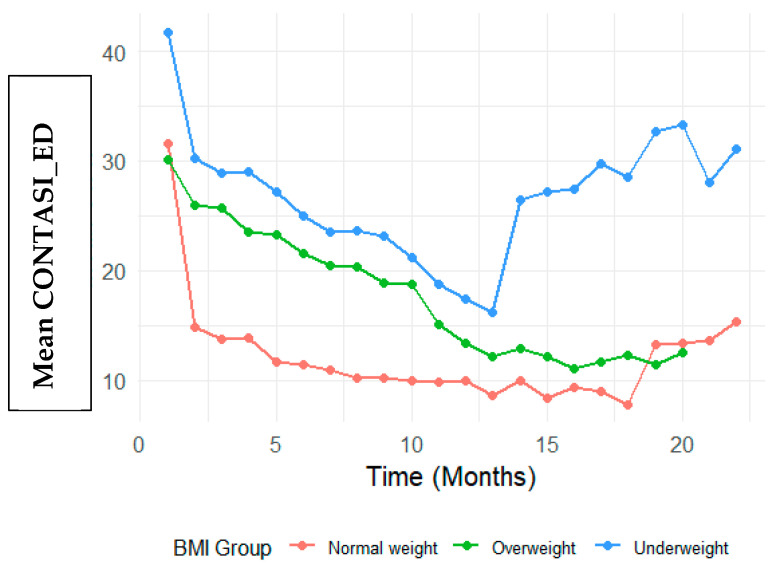
Differences in CONTASI-ED based on BMI group.

**Figure 3:**
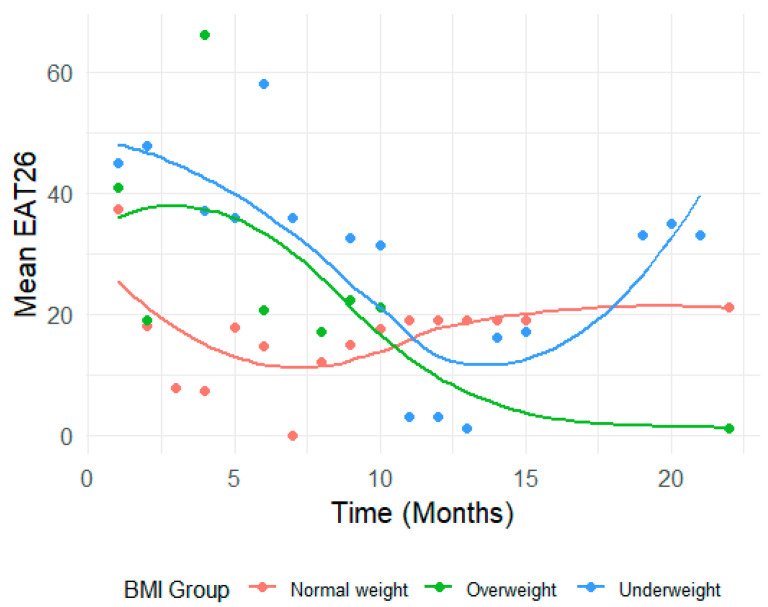
Differences in EAT-26 scores based on BMI group.

Convergent validity: Convergent validity was assessed by examining correlations between the CONTASI-ED and EAT-26 scores over the course of treatment. The CONTASI-ED scores were strongly correlated with EAT-26 scores throughout treatment (Figure 2 and Figure 3). Initially, both measures were high, gradually decreasing over the course of treatment, until around 12 months—after which occasional regressions were observed. These regressions initially reflected the disclosure of previously hidden symptoms (such as restriction, purging or compulsive physical activity) and subsequent shifts in eating disorder subtypes (e.g., from restrictive anorexia nervosa to binge–purge anorexia or bulimia nervosa).

Internal consistency: Internal consistency was calculated using McDonald’s Omega, accounting for different item loadings and variations in the strength of the items’ associations with the construct being measured [36]. Apart from the “Start” category, all other categories exhibit omega coefficients above the acceptable threshold of 0.70—ranging between 0.72 and 0.9.

### 3.3. Change in CONTASI-ED Scores over Time

The CONTASI-ED scores decreased significantly over time. From Time 1 to Time 2, scores decreased by 12.75 units (B = −12.75, 95% CI: −15.12 to −10.39). By Time 13, the cumulative reduction reached 20.87 units (B = −20.87, 95% CI: −23.80 to −17.94).

Occasional score increases at later time points were observed, particularly among participants with extended treatment duration. While these patterns may reflect temporary symptom exacerbation, they could also indicate increased insight or willingness to disclose previously unreported symptoms. As this interpretation is speculative, further research is needed to clarify whether such score elevations reflect true clinical worsening or a shift in self-awareness and reporting.

#### BMI Group Differences

At baseline, Underweight participants had significantly higher CONTASI-ED scores than the Normal Weight group (B = 10.04, 95% CI: 2.86 to 17.21, *p* = 0.006). The Overweight group did not significantly differ from the Normal Weight group (B = −1.32, 95% CI: −10.51 to 7.87, *p* = 0.778) (Figure 2). Visual inspection of Figure 2 reveals distinct patterns of symptom reduction by BMI group. While all groups showed declining CONTASI-ED scores over time, the Underweight group exhibited a more gradual descent with a flatter slope in early treatment phases—suggesting potential resistance or slower response. In contrast, participants in the Overweight group demonstrated steeper initial declines, indicating a more rapid symptom reduction in the early weeks. The Normal Weight group displayed a more linear and consistent downward trend. These visual trends complement the model-based findings and underscore the clinical heterogeneity in treatment response trajectories.

Interaction effects indicated that Underweight participants showed a slower rate of improvement in the early stage of treatment. For example, at Time 2, the difference in reduction was significantly smaller (Time 2: B = 9.19, 95% CI: 3.98 to 14.39, *p* = 0.001). By Time 8, however, this difference diminished and was no longer statistically significant (B = 3.67, 95% CI: −1.67 to 9.01, *p* = 0.177).

Conversely, participants in the Overweight participants demonstrated greater reductions in the CONTASI-ED scores than those of the Normal Weight group, with significant interaction effects at multiple time points (e.g., Time 2: B = 11.08, 95% CI: 4.16 to 17.99, *p* = 0.002).

### 3.4. Change in EAT-26 Scores over Time

EAT-26 scores also showed significant reductions across time (*p* < 0.001). From baseline to Time 2, scores dropped by 14.77 units (B = −14.77, 95% CI: −21.01 to −8.54). By Time 10, the total reduction reached 22.13 units (B = −22.13, 95% CI: −28.79 to −15.46).

In the Underweight group, interaction effects indicated limited changes in EAT26 scores over time. While early time points suggested larger increases (e.g., Time 2: B = 9.85, 95% CI: −5.32 to 25.02, *p* = 0.201), these trends were not statistically significant and diminished further at subsequent time points (e.g., Time 10: B = 2.80, 95% CI: −14.01 to 19.62, *p* = 0.742).

In contrast, the Overweight group showed significant increases in EAT-26 scores at specific time points—notably at Time 4 (B = 33.54, 95% CI: 6.59 to 60.49, *p* = 0.015) (Figure 3). As shown in Figure 3, the Overweight group exhibited fluctuations in EAT-26 scores with notable spikes (e.g., at Time 4), possibly reflecting episodic changes in cognitive attitudes toward eating. The Underweight group showed relatively flat trajectories, with limited downward movement, indicating a potential disconnect between behavioral and cognitive symptom improvement. These visual patterns illustrate the differential sensitivity of EAT-26 across subgroups and reinforce the importance of multidimensional assessment tools.

However, these patterns were inconsistent over time, with many time points yielding non-significant interaction effects (e.g., Time 9: B = 4.86, 95% CI: −12.22 to 21.94, *p* = 0.574).

### 3.5. Multivariable Analysis

#### 3.5.1. Effect of Age on CONTASI-ED Scores

Age was significantly associated with higher CONTASI-ED scores in the Overweight group (B = 1.42, 95% CI: 0.36 to 2.49, *p* = 0.010), but only to a limited degree with Underweight and Normal Weight participants.

Including age as a covariate improved model fit for the Overweight group, with the marginal R^2^ increasing from 0.437 to 0.604 (Supplementary Materials S2a).

#### 3.5.2. Effect of PTSD on CONTASI-ED Scores

Adjusting for PTSD had a more pronounced effect on the Normal Weight participants. In the Underweight group, PTSD had no significant impact (B = 1.42, 95% CI: −13.14 to 15.98, *p* = 0.848), and baseline CONTASI-ED scores remained stable (41.48, 95% CI: 34.94 to 48.02) (Supplementary Materials S2b).

Similarly, in the Overweight group, PTSD was not a significant predictor (B = 8.89, 95% CI: −1.85 to 19.63, *p* = 0.102), and its inclusion did not substantially change baseline CONTASI-ED estimates (30.12, 95% CI: 23.36 to 36.89). However, in the Normal Weight group, PTSD was strongly associated with lower CONTASI-ED scores (B = −16.81, 95% CI: −22.50 to −11.12, *p* < 0.001), and adjusting for PTSD increased the baseline estimate from 33.99 (95% CI: 28.29 to 39.70) to 31.45 (95% CI: 27.28 to 35.61). This stratified analysis was exploratory and intended to probe potential variation in comorbidity patterns, although the observed effects should be interpreted with caution due to limited subgroup sizes.

## 4. Discussion

This study provides initial validation of the CONTASI-ED as a psychometrically sound and clinically responsive tool for assessing eating disorder (ED) severity and tracking treatment progress. Designed to address key limitations of existing instruments, the CONTASI-ED integrates state-based indicators (e.g., physiological markers) with trait-level factors (e.g., trauma history, compulsivity), offering a holistic and nuanced assessment framework.

The CONTASI-ED demonstrated strong test–retest and inter-rater reliability, comparable to or exceeding those reported for established instruments such as the EDE-Q [19] and EPSI-CRV [3]. This suggests that the tool yields stable and consistent assessments across raters and time, even in varied clinical settings. Moreover, the strong alignment between clinician- and patient-rated scores reinforces its applicability as a dual-mode instrument, adaptable to clinician or self-report formats depending on patient needs.

The CONTASI-ED also demonstrated high internal consistency for most categories, supporting its structural coherence. As anticipated, the “Start” category yielded lower omega values due to its broader coverage of historical risk factors—consistent with psychometric literature suggesting reduced reliability in multidomain constructs [36].

Importantly, the CONTASI-ED effectively differentiated between ED patients and healthy controls providing evidence for its discriminant validity. This aligns with previous literature establishing large effect sizes for tools like EAT-26 in distinguishing clinical from non-clinical groups [18], but the present results suggest even greater sensitivity, potentially due to the inclusion of both behavioral and physiological domains.

One of the most notable findings was the CONTASI-ED’s sensitivity to symptom change over time. Scores decreased significantly across treatment phases, with occasional increases interpreted as reflections of greater honesty and insight. These “blips” may reflect either temporary symptom exacerbation or increased trust in the therapeutic alliance, leading to greater disclosure of previously unreported symptoms. However, this interpretation remains speculative and should be further examined in future research before being considered indicative of ‘authentic worsening’ or deeper self-awareness—paralleling prior work suggesting that symptom disclosure can transiently elevate scores without indicating relapse [33]. In contrast, EAT-26 scores remained flatter, missing these nuanced shifts—suggesting that the CONTASI-ED may be better equipped to capture clinically meaningful fluctuations than static screening tools.

Group-based analyses offered further insight. Underweight participants had higher baseline scores and showed slower early improvement compared to those at normal weight, consistent with prior studies indicating that low BMI is associated with more entrenched cognitions and treatment resistance [26]. In contrast, participants with overweight status demonstrated greater symptom reduction, potentially reflecting responsiveness to interventions addressing binge-eating patterns [5].

Multivariable analyses revealed additional complexity. Older age was associated with higher severity in the Overweight group, suggesting that chronicity may moderate treatment response in this subgroup. PTSD was significantly associated with symptom severity only in the Normal Weight group—a finding that reinforces the importance of trauma-informed assessment tools. One possible explanation is that individuals in this weight category may experience fewer external indicators of illness, leading to an increased role of internalized emotional distress in symptom expression. PTSD-related hypervigilance, avoidance, or dissociation could interfere with treatment responsiveness or amplify underlying compulsive behaviors. These interpretations remain exploratory and warrant targeted investigation in future studies. This finding also echoes prior work on the prognostic role of comorbidity in ED outcomes [4,13].

In light of emerging research on emotion dysregulation as a central mechanism in eating pathology, it is notable that several CONTASI-ED domains—particularly Compulsiveness, Self-Care, and the inclusion of mood and anxiety indicators—may indirectly capture aspects of dysregulation. For example, patterns of impulsive behaviors, sleep disruption, and restrictive eating cycles often reflect difficulties in emotional containment. While the tool was not explicitly designed to quantify emotion regulation capacity, future work could explore how specific subdomains of the CONTASI-ED correlate with validated emotion dysregulation scales, thereby enriching its clinical interpretability and relevance to transdiagnostic processes, a finding that reinforces the importance of trauma-informed assessment tools and echoes prior work on the prognostic role of comorbidity in ED outcomes [4,13]. The CONTASI-ED also demonstrated clear advantages in routine clinical application. Its flexible design enables tracking of progress across settings—from inpatient to outpatient—without losing interpretive continuity. While not a focus of the present study, the modular structure and dual-mode administration of the CONTASI-ED may lend itself to future digital implementation. This possibility remains untested in our current data and is proposed solely as a direction for future research, particularly in the context of stepped-care models and remote monitoring strategies.

While the CONTASI-ED was designed with contextual adaptability in mind—including phrasing that allows for clinical and cultural tailoring—this flexibility has not yet been empirically tested. The current study was conducted in a culturally homogenous sample, limiting the ability to assess cross-cultural validity. Future studies should examine whether the tool performs equivalently across diverse populations and healthcare systems.

Unlike static tools with cutoff scores, the CONTASI-ED allows clinicians to monitor progress on a continuous scale, capturing meaningful intra-individual variation and informing real-time decisions. This approach is consistent with the rationale behind tools such as the CR-EAT, which emphasizes session-by-session tracking rather than fixed thresholds [15]. In our clinical observations, reductions in the CONTASI-ED scores below 10 coincided with substantial functional improvement and justified a step-down in treatment intensity—a pattern that warrants future empirical validation.

Finally, the inclusion of objective physiological markers such as bradycardia and electrolyte abnormalities further distinguishes the CONTASI-ED from existing tools. These markers not only improve face validity and clinical confidence but also facilitate communication across interdisciplinary teams (e.g., psychiatry, internal medicine, nutrition)—a key requirement in ED care settings [28].

### Limitations and Future Research Directions

While the present findings support the CONTASI-ED’s strong psychometric properties and clinical applicability, several limitations warrant consideration. First, the sample was predominantly female and relatively homogenous in terms of cultural background, which limits the generalizability of the findings to males, non-binary individuals, and more diverse populations. This limitation is common in ED research but highlights the importance of validating assessment tools in underrepresented groups [32] Further studies should include broader demographic samples to assess measurement invariance across sex and cultural background.

Second, although the CONTASI-ED was designed to capture a wide range of behavioral, cognitive, and physiological indicators, certain contextual and protective factors—such as social connectedness, body appreciation, or self-compassion—were not included. These factors may play a critical role in moderating ED severity and predicting recovery trajectories [37], and their exclusion may limit the tool’s comprehensiveness. Incorporating such elements into future revisions or as parallel modules may enhance its predictive value. Moreover, future studies may want to validate each subdomain with specific tools—particularly constructs such as emotion regulation and compulsivity.

Third, although domain weights were informed by both expert consensus and factor analysis, their empirical grounding remains preliminary. For example, obsessive cognitions were weighted more heavily than some physiological markers, based on the clinical assumption that they are more resistant to change and more strongly associated with relapse risk. While this reflects established clinical reasoning [3], it may also introduce a theoretical risk that multidimensional scoring may inadvertently over-pathologize certain subgroups—especially when psychosocial variables are weighted alongside physiological indicators. Future large-scale predictive modeling could help refine the weighting schema and ensure that clinical relevance does not come at the expense of diagnostic inflation.

Additionally, the relatively modest sample size and the naturalistic setting of the study, while ecologically valid, may limit statistical power and preclude certain subgroups or longitudinal comparisons. Larger samples across multiple sites would allow for more robust analyses of measurement invariance, predictive validity, and sensitivity to treatment phase or subtype transitions.

Finally, it is important to note that the “Social and/or mentalizing impairment” item category was added after the primary analyses were conducted. Thus, it was excluded from all quantitative analyses reported in this study and is not reflected in any of the psychometric evaluations. While this domain reflects a growing interest in social-cognitive factors in EDs [23], its psychometric contribution and predictive utility should be evaluated in future studies before being integrated into the core scoring algorithm.

## 5. Conclusions

The CONTASI-ED represents a novel, multidimensional assessment tool that bridges key gaps in ED evaluation—combining clinical depth, time efficiency, and sensitivity to therapeutic change. Its adaptability across care settings, robust psychometric properties, and incorporation of physiological and psychosocial indicators position it as a valuable addition to the measurement-based care landscape.

As mental health systems move toward more personalized, data-informed practices, tools like the CONTASI-ED offer a scalable and clinically meaningful way to guide decision-making and improve treatment outcomes across the diverse spectrum of eating disorders.

## Figures and Tables

**Table 1:** Structure and scoring of the final version of the assessment tool.

Domain	Number of Subcategories	Number of Items	Max Score	Weight Key Indicators *	Example of Factors Included
**Starting Point**	6	11	22	22%	Previous treatments; occupation status; trauma history; substance use
**Anthropometrics and Menstrual Cycle**	4	4	16	16%	Weight trajectory; weight stability; menstrual status
**Pathophysiology**	3	19	13	13%	Bradycardia; orthostatic; hypotension; gastrointestinal symptoms; blood test
**Self-Care**	4	4	16	16%	Sleep hygiene; general self-care behaviors; physical activity patterns; medication adherence
**Compulsiveness**	4	22	16	16%	Eating restriction; over-controlling; other compulsive behaviors; use of laxatives or vomiting
**Obsessiveness**	7	20	18	18%	Intrusive thoughts; cognitive rigidity

* WKI = Max category score/max points.

**Table 2:** The sample demographic and clinical characteristics.

Characteristic	Diagnosed with ED		Healthy Participants
**Setting of treatment**	Outpatient clinic *N* = 48	Intensive day clinic *N* = 10	*N* = 10
Age (mean ± SD)	19.7 (SD = 6.4)	24.0 (SD = 3.2)	20 (SD = 5.5)
BMI at admission	20.0 (SD = 5.7)	19.66 (SD = 6.1)	23.4 (SD = 6.8)
Education	11	12	13
ED diagnosis at admission	Restrictive AN 24 (50%) Binge Purge AN 5 (10%) Atypical AN 7 (14.6%) BN 7 (14.6%) BED 5 (10.5%)	Restrictive AN 7 (70%) Binge Purge AN 3 (30%)	–
PTSD	32 (55%)	2 (20%)	–

**Table 3:** Comparison of CONTASI-ED and EAT-26 scores between diagnosed and healthy participants.

Variable	Overall *N* = 58 ^1^	Diagnosed *N* = 17 ^1^	Healthy *N* = 41 ^1^	*p*-Value ^2^
**STATT1**	26.7 (14.9)	7.6 (4.7)	34.6 (9.4)	<0.001
**Eat26T1**	35.8 (19.0)	5.7 (3.4)	40.2 (16.0)	<0.001
**Unknown**	11	11	0	

^1^ Mean (SD) and ^2^ Welch two sample *t*-test.

## Data Availability

The dataset supporting the conclusions of this article is available from the corresponding author upon reasonable request. Due to concerns about potential re-identification despite anonymization, and to protect participant confidentiality, the dataset will not be publicly uploaded at this stage.

## Supplementary Materials

The following supporting information can be downloaded at: https://www.mdpi.com/article/10.3390/nu17111790/s1, Supplementary Materials S1—the questionnaire; Supplementary Materials S2—table of statistics.
