# Supplementary material for: The Conclusive and Continuous Tool to Assess Severity and Improvement of Eating Disorders (CONTASI-ED): Development and Psychometric Properties
Source: Nutrients. 2025 May 24;17(11):1790. doi: 10.3390/nu17111790 (PMC12157281; doi:10.3390/nu17111790)
Supplement: Supplementary file 1 [file nutrients-17-01790-s001.zip › nutrients-3630555-Supplementary Materials .pdf]

Supplementary Materials S1—The questionnaire

Personal Information

Full Name______________________ ID _______________ Date of Birth ____________ Age______
Mobile_______________________ Email________________ Address: _________________________
Occupation__________________ Ethnicity________________ Rate of current food insecurity (0-3) ____

---

1. Starting Point

Item	Options	Score	Admission score	Date of scoring
Previous ED Treatments	1st, 2nd, 3rd	0	___	
	4th, 5th	1		
Number of psychiatric or ED hospitalizations				
	0	0	____	____
	1	1		
	>1	2		
Trauma History				
	Childhood maltreatment	1	___	
	Social trauma, bullying	`1	___	
	Sexual abuse	2	___	____
	Presence of flashbacks	2	____	
Other risk conditions such as				
	ASD or other mental illness	2	____	____
	OCD (not related to ED)	2	____	
Addictions				
	Withdrawal for > 2 years	1	____	
	Recurrent episodes of loss of control due to drunkenness	1	____	____
	Smoking drugs beyond social events	2		
Current employment status	Full-time	0		
	Part-time due to illness	2		
Occupational impairment (school, work, other) due to illness		4	____	____
**Sum of 11 items total scores**	**Max score 22**			
The last item in the bottom of this section was added after the current analysis.				
**Social &/or mentalizing impairment***	Prior to illness	2		
**Sum of 12 items total scores**	**Max score 24**		____	____

* social and/or mentalization impairment prior to illness was added after analysis

2. Weight and menstrual cycle

Current height (cm)____ Minimum weight ______date______ Maximum weight ______date______
Pre-disorder Weight__________ Pre-disorder BMI or BMI percentile_______ Agreed target weight______
_

Item	Options	Score	Admission Score	Date of assessment
**Weight (kg)___ Height (cm) ____ BMI__**	BMI>40	4		
	BMI>35	3		
	BMI>30	2		
	BMI>27	1		
	BMI > 20	0		

	BMI 18.1 – 19.9	1		
	BMI 17.1 – 18.0	2		
	BMI 14.1 – 16.9	3		
	BMI < 14	5	____	____
**Under age of 20**				
	> 5th and under pre ED percentage	2		
	3-5th BMI percentage	3		
	1-3th BMI percentage	4		
	Below 1st percentage	5	____	____
Percent of Weight Lost				
	**5%** weight loss	**0**		
	**10%**	**1**		
	**15%**	**2**		
	**20%**	**3**		
	**25%**	**4**	____	____
	**30%**	**5**		
Weight stability				
In a state of poor weigh stability	6 months	1		
	12 months	2		
	18-23 months	3		
	Longer	4		
In target weight range	6 months	0		
	12 months	-1	____	____
	18 months and more	-2		
Menstrual cycle				
Regular		0		
Primary amenorrhea, or irregular/low menstrual flow upon return		1		
Secondary amenorrhea		2	____	____
**Sum of 4 items total scores**	**MAX score: 16**		____	____

3. Pathophysiology

**Item**	**Options**	**Score**	**Admission score**	**Date of scoring**
Ingestive system Diarrhea, constipation, nausea (unrelated to usage of drugs)	0			
Emetophobia	+ 1	____		
Compulsive bowel control	+ 1	____		
Diagnosed inflammatory bowel disease (IBD)	+ 2	____	____	____
Other physical signs				
Exhaustion<br>Dizziness/weakness during exertion<br>Difficulty concentrating<br>General exhaustion<br>Shortness of breath<br>Panic attacks	2-3 Symptoms	+ 1		
	4-6 Symptoms	+ 2		

Sensitivity to cold, blue lips or fingers<br>Depression<br>Sleep disturbances<br>Hand tremors<br>Lanugo (fine body hair growth)<br>Bone density issues<br>Other: _______________	7-9 Symptoms	+ 3	____	____
	> 10 Symptoms	+ 4		
Blood and Heart Test Findings				
ECG abnormalities<br>- prolonged QTc interval<br>- severe bradycardia (adult: HR⩽30 bpm; adolescent: HR⩽45 bpm))<br>- severe hypotension: ⩽90/45 mm Hg<br>- orthostatic intolerance (a supine to standing systolic BP drop>20 mm Hg and a diastolic drop>10 mm Hg)<br>- arrhythmias<br>- pulse under 50 bpm<br>Fasting glucose <70 mg/dL<br>Blood counting<br>- Hemoglobin <11 (g/dL)<br>- Ferritin (ng/mL) <10<br>- MCV (fl/cell) <76<br>- MCH (pg/RBC) <27<br>- MCHC (g/dL) <33<br>- */WBC ($10^3/\mu L$)* <4.6<br>Electrolyte disturbances<br>- Potassium (K, mmol/L)<3.5<br>- Phosphorus (P, mg/dL)< 3<br>- Magnesium (Mg, mg/dL) <1.9<br>Sodium (Na, mmol/L) >135<br>TSH<0.5 mIU/L, T4<10 pmol/L<br>Amylase (U/L) >100	One moderate pathological subcategory	0	____	____
	Severe one pathological sign	2		
	Two moderate pathological subcategories	2		
	Two severe pathological subcategories	3		
	Three or more pathological subcategories	4		
	Refeeding syndrome or water intoxication	5		
**Sum of 19 items total Scores**	**MAX score: 13**		____	____

4. Self-Care

Item	Options	Score	dmission score	Date of scoring
Sleep Hygiene				
No issues < than 0.5 hr. to fall a sleep		0	____	____
Minor disturbances:<br>Presence of nightmares and/or takes more than 0.5 hrs. to fall asleep or 1-2 wake ups during night		1		
Moderate disturbances:<br>Presence of nightmares & takes more than 1 hrs. to fall asleep or more than 2 wake ups during night.		2		
Severe disturbances, chronic insomnia and day tiredness		3		
General self-care behaviors:<br>1. Maintains routine of sleep hygiene<br>2. Maintains a structured eating routine<br>3. Usually respond to hunger and satiety cues<br>4. Maintains routine of self-hygiene<br>5. Maintains routine of healthy physical activity (2-4 times per week)<br>6. Uses sun protection and wears a hat when exposed to the sun				

	Usually maintains all 5-6	0		
	Usually maintains 3-4 out of 6	1		
	Usually maintains 2-3 out of 6	2		
	Usually maintains less than 2	4	_____	_____
Responsibility regarding medicines				
	90-100% of time	0		
	80-89% of time	1		
	60-70% of time	2		
	50% of time	3		
	Refuses although needed	4	_____	_____
Eating habits				
Selective eating (rather diverse eating)	0-3 items	0		
Nibbling (rather than having full meals), with or without snacks				
**Excessive control:** Ignoring hunger cues, demanding environmental control				
Skipping breakfast most days				
Skipping lunch most days				
Skipping dinner most days	4-7 items	1		
Stop eating early (before 18:00)				
Ignoring/denying/refusing to have snacks				
Extreme avoidance of fatty foods or sweets				
Rigid eating (set times, limited variety, strict preparation methods) rather than flexible and spontaneous eating	8-11 items	2		
Stop eating when possible, rather than when actually full, tendency to eat too little				
Creating a "food control" atmosphere at home (banning kitchen access while eating, no cooking, no guests, etc.)				
Demanding special food preparation (rather than eat what everyone else eats and what is available).	12-16 items	3		
**Lack of control :** Ignoring fullness and satiety cues (difficulty stopping and/or delay gratifications)				
Impulsivity and mindless eating (rather than – mindful eating and minded response to stimulus)				
Consuming sweets and snacks instead of real foods. Eat mainly "children's food" (fries, schnitzel, sweets)	17-21 items	4		
Eating while standing or from the pot (rather than while sitting and eating from personal plate)				
Eating while doing another activity (watching TV, reading, etc. rather than just eating (alone or with others)				
Night eating (after going to bed or during late\evening due to sleep difficulties				
**Miscellaneous**				
Eating in the bedroom/living room rather than in the dining area/kitchen				
At a restaurant, tasting from others rather than ordering a separate dish	> 21 items	5		
Eating only alone vs. Eating socially and participating in family meals				
Eating very fast or very slow				
Consuming a large amount of diet products				

Messy, unrefined eating rather than aesthetic			____	____
Wearing oversized/baggy clothes to hide body				
**Sum of 4 items' total scores**	**MAX:16**		____	____

5. Compulsive Behaviors

Item	Options	Score	Admission score	Date of scoring
**Compensatory behaviors**				
Laxative use		1	____	____
Compulsive exercising		1	____	____
Chewing and spitting out food		1	____	____
Self-induced vomiting (days per week)	2-3 per week	2	____	____
	4-6	3		
	> 7 times per week	4	____	____
Compulsive binge eating episodes		2	____	____
**Non-suicidal self-injury behaviors (NSSI)**	No=0 Yes=2	2	____	____
**Food avoidance/restrictions rituals**				
Calorie counting	2-3 items	1		
Consumption of low-calorie products				
Crumbling food while eating, mashing food, leaving food on the plate				
Throwing away food and pretending it was eaten				
Excessive reheating, delaying meals (distracting conversations, stalling before eating)	4-7 items	2		
Excessive beverage consumption (caffeine, water, diet drinks)				
Avoiding "forbidden" foods				
Excessive chewing of gum	8-11 items	3		
Prolonged meal time >30 min				
Bite counting				
Eating only alone				
Cooking, baking, and feeding others without eating oneself	12-15 items	4		
**Repeating checking**				
Repeated questions as an expression of difficulty taking responsibility: "Do I have to eat?"				
Body checking (frequent body scrutiny for changes)			____	____
Compulsive food tracking or compulsive photographing of meals vs. Relevant tracking	>16 items	5		
Frequent self-weighing				
**Sum of 22 total scores**	**MAX 16**		____	____

6. Obsessive Thoughts/Perceptions

Item	Score	Admission Score	Date of assessment
Body dysmorphia			
Drive for body weight redistribution or recompositing	1		
Drive for muscularity (pursuit of a muscular appearance)	1		
Body image distortion (perceiving certain body parts are distorted)	1		
Unbearable feeling of fullness	1	____	
Rigidity regarding an ideal appearance			
Rigidity regarding a specific weight	1		
Pursuit of thinness	1	____	
Fear of weight gain	1		
Ambivalence about ED recovery, perceiving it as anti-obesity protector	1		
Food preoccupation			
"I don't deserve it."	1	____	
Fear of eating	1		
Feeling guilty after eating	1		
Fear of losing control over eating ("a bottomless pit")	1		
Compensatory behaviors			
The food/calories can't stay in my body	1	____	
"I must exercise intensely for at least 1.5 hours per day to deserve normal eating or social participation.".	1	____	
Cognitive Rigidity in Self-Appraisal			
Tendency to assess oneself in a rigid, all-or-nothing manner, leading to recurring thoughts of punishment or rewards	1	____	
Catastrophizing failure in various fields	1		
**Excessive need to control situations and people**			
Obsessive need to control situations & people	1	____	
The rule of obsessive precision	1		
**General repetitive thinking due to general anxiety**	1	____	
**Difficulty letting go of the "sick" status** that legitimizes certain failures or weaknesses and provide secondary profits	1	____	
**Sum of total 20 items scores MAX 18**		____	____

Supplementary Materials S2— Table of statistics

Supplement 2.a. Effect of age on CONTASI-ED scores across BMI groups using linear mixed-effects models

	**Underweight**			**Normal weight**			**Overweight**		
Predictors	*B*	*CI*	*p*	*B*	*CI*	*p*	*B*	*CI*	*p*
(Intercept)	39.75	10.75 – 68.76	**0.008**	23.82	11.84 – 35.80	**<0.001**	0.16	-22.84 – 23.17	0.989
Time [2]	-7.60	-11.81 – -3.40	**<0.001**	-16.86	-20.05 – -13.67	**<0.001**	-5.91	-10.41 – -1.42	**0.011**
Time [3]	-8.91	-13.11 – -4.71	**<0.001**	-17.89	-21.08 – -14.70	**<0.001**	-8.18	-13.13 – -3.23	**0.002**
Time [4]	-9.85	-13.91 – -5.78	**<0.001**	-17.92	-21.16 – -14.67	**<0.001**	-8.29	-12.79 – -3.79	**0.001**
Time [5]	-11.65	-15.71 – -7.58	**<0.001**	-19.38	-22.63 – -16.13	**<0.001**	-6.78	-10.97 – -2.59	**0.002**
Time [6]	-13.90	-17.96 – -9.83	**<0.001**	-20.31	-23.61 – -17.00	**<0.001**	-8.48	-12.67 – -4.29	**<0.001**
Time [7]	-15.32	-19.39 – -11.25	**<0.001**	-20.65	-23.96 – -17.34	**<0.001**	-9.58	-13.77 – -5.39	**<0.001**
Time [8]	-17.34	-21.69 – -12.98	**<0.001**	-21.03	-24.23 – -17.83	**<0.001**	-9.83	-13.81 – -5.86	**<0.001**
Time [9]	-17.74	-22.10 – -13.39	**<0.001**	-21.37	-24.62 – -18.12	**<0.001**	-11.25	-15.22 – -7.28	**<0.001**
Time [10]	-19.77	-24.13 – -15.42	**<0.001**	-22.36	-25.96 – -18.75	**<0.001**	-11.42	-15.39 – -7.44	**<0.001**
Time [11]	-20.41	-25.97 – -14.85	**<0.001**	-20.90	-24.86 – -16.95	**<0.001**	-10.55	-15.53 – -5.58	**<0.001**
Time [12]	-21.66	-27.22 – -16.10	**<0.001**	-20.73	-24.68 – -16.78	**<0.001**	-12.22	-17.19 – -7.24	**<0.001**
Time [13]	-22.91	-28.47 – -17.35	**<0.001**	-22.08	-26.03 – -18.13	**<0.001**	-13.47	-18.44 – -8.49	**<0.001**
Time [14]	-20.70	-25.53 – -15.87	**<0.001**	-20.68	-24.63 – -16.73	**<0.001**	-12.80	-17.78 – -7.83	**<0.001**
Time [15]	-20.66	-25.49 – -15.83	**<0.001**	-22.50	-26.79 – -18.22	**<0.001**	-13.55	-18.53 – -8.58	**<0.001**
Time [16]	-22.53	-27.70 – -17.35	**<0.001**	-22.39	-27.18 – -17.59	**<0.001**	-12.56	-20.17 – -4.95	**0.002**

Time [17]	-19.73	-24.90 – -14.55	**<0.001**	-22.80	-27.60 – -18.01	**<0.001**	-11.52	-17.28 – -5.77	**<0.001**
Time [18]	-21.23	-26.40 – -16.05	**<0.001**	-23.30	-28.45 – -18.15	**<0.001**	-10.90	-16.65 – -5.15	**<0.001**
Time [19]	-22.18	-27.83 – -16.53	**<0.001**	-18.38	-23.16 – -13.60	**<0.001**	-11.77	-17.53 – -6.02	**<0.001**
Time [20]	-24.82	-32.29 – -17.35	**<0.001**	-18.29	-23.07 – -13.51	**<0.001**	-10.24	-17.89 – -2.59	**0.010**
Time [21]	-26.95	-34.32 – -19.58	**<0.001**	-18.61	-25.01 – -12.21	**<0.001**			
Time [22]	-23.63	-29.91 – -17.35	**<0.001**	-17.46	-23.10 – -11.82	**<0.001**			
Age	0.09	-1.32 – 1.50	0.904	0.40	-0.19 – 0.98	0.183	1.42	0.36 – 2.49	**0.010**
**Random Effects**									
σ^2	22.63			26.56			11.74		
τ_{00}	119.24 $_{Number}$			65.64 $_{Number}$			24.53 $_{Number}$		
ICC	0.84			0.71			0.68		
N	12 $_{Number}$			21 $_{Number}$			6 $_{Number}$		
Observations	144			264			73		
Marginal R^2 / Conditional R^2	0.249 / 0.880			0.309 / 0.801			0.604 / 0.872		

Supplement 2.b. Linear mixed-effects models for CONTASI-ED scores over time with and without adjustment for covariates

	Model 1			Model 2		
Predictors	*B*	*CI*	*p*	*B*	*CI*	*p*
(Intercept)	34.38	30.75 – 38.01	**<0.001**	31.44	27.13 – 35.76	**<0.001**
Time [2]	-12.75	-15.12 – -10.39	**<0.001**	-16.86	-19.86 – -13.86	**<0.001**
Time [3]	-13.99	-16.38 – -11.60	**<0.001**	-17.88	-20.88 – -14.89	**<0.001**

Time [4]	-14.11	-16.47 – -11.74	**<0.001**	-17.91	-20.96 – -14.86	**<0.001**
Time [5]	-15.27	-17.62 – -12.93	**<0.001**	-19.36	-22.41 – -16.30	**<0.001**
Time [6]	-16.71	-19.08 – -14.34	**<0.001**	-20.30	-23.40 – -17.19	**<0.001**
Time [7]	-17.55	-19.92 – -15.18	**<0.001**	-20.64	-23.75 – -17.53	**<0.001**
Time [8]	-18.24	-20.59 – -15.90	**<0.001**	-21.02	-24.02 – -18.01	**<0.001**
Time [9]	-18.81	-21.18 – -16.45	**<0.001**	-21.37	-24.42 – -18.31	**<0.001**
Time [10]	-19.98	-22.48 – -17.48	**<0.001**	-22.35	-25.74 – -18.96	**<0.001**
Time [11]	-19.08	-22.01 – -16.15	**<0.001**	-20.93	-24.64 – -17.21	**<0.001**
Time [12]	-19.56	-22.49 – -16.63	**<0.001**	-20.75	-24.47 – -17.04	**<0.001**
Time [13]	-20.87	-23.80 – -17.94	**<0.001**	-22.10	-25.82 – -18.39	**<0.001**
Time [14]	-19.35	-22.17 – -16.53	**<0.001**	-20.70	-24.42 – -16.99	**<0.001**
Time [15]	-20.42	-23.36 – -17.49	**<0.001**	-22.54	-26.56 – -18.51	**<0.001**
Time [16]	-20.92	-24.27 – -17.56	**<0.001**	-22.44	-26.95 – -17.94	**<0.001**
Time [17]	-19.80	-23.06 – -16.55	**<0.001**	-22.86	-27.36 – -18.35	**<0.001**
Time [18]	-20.45	-23.80 – -17.10	**<0.001**	-23.35	-28.19 – -18.51	**<0.001**
Time [19]	-18.40	-21.75 – -15.05	**<0.001**	-18.43	-22.92 – -13.93	**<0.001**

Time [20]	-18.07	-21.82 – -14.32	**<0.001**	-18.34	-22.84 – -13.85	**<0.001**
Time [21]	-20.66	-25.47 – -15.85	**<0.001**	-18.66	-24.68 – -12.65	**<0.001**
Time [22]	-18.58	-22.75 – -14.41	**<0.001**	-17.51	-22.81 – -12.21	**<0.001**
BMI group [Overweight]				-1.32	-10.51 – 7.87	0.778
BMI group [Underweight]				10.04	2.86 – 17.22	**0.006**
Time [2] × BMI group [Overweight]				11.08	4.16 – 17.99	**0.002**
Time [3] × BMI group [Overweight]				9.79	2.31 – 17.28	**0.010**
Time [4] × BMI group [Overweight]				9.75	2.82 – 16.69	**0.006**
Time [5] × BMI group [Overweight]				12.63	6.07 – 19.19	**<0.001**
Time [6] × BMI group [Overweight]				11.87	5.28 – 18.46	**<0.001**
Time [7] × BMI group [Overweight]				11.12	4.53 – 17.71	**0.001**
Time [8] × BMI group [Overweight]				11.19	4.91 – 17.46	**0.001**
Time [9] × BMI group [Overweight]				10.12	3.82 – 16.41	**0.002**

Time [10] × BMI group [Overweight]	10.93	4.47 – 17.40	**0.001**
Time [11] × BMI group [Overweight]	10.16	2.33 – 17.99	**0.011**
Time [12] × BMI group [Overweight]	8.32	0.49 – 16.15	**0.037**
Time [13] × BMI group [Overweight]	8.42	0.59 – 16.25	**0.035**
Time [14] × BMI group [Overweight]	7.68	-0.14 – 15.51	0.054
Time [15] × BMI group [Overweight]	8.77	0.79 – 16.75	**0.031**
Time [16] × BMI group [Overweight]	9.77	-1.69 – 21.24	0.095
Time [17] × BMI group [Overweight]	11.15	2.00 – 20.30	**0.017**
Time [18] × BMI group [Overweight]	12.27	2.94 – 21.59	**0.010**
Time [19] × BMI group [Overweight]	6.47	-2.68 – 15.61	0.165
Time [20] × BMI group [Overweight]	7.85	-3.67 – 19.36	0.181
Time [2] × BMI group [Underweight]	9.19	3.98 – 14.39	**0.001**

Time [3] × BMI group [Underweight]	8.91	3.70 – 14.11	**0.001**
Time [4] × BMI group [Underweight]	8.01	2.89 – 13.13	**0.002**
Time [5] × BMI group [Underweight]	7.66	2.53 – 12.78	**0.004**
Time [6] × BMI group [Underweight]	6.35	1.19 – 11.51	**0.016**
Time [7] × BMI group [Underweight]	5.27	0.11 – 10.43	**0.045**
Time [8] × BMI group [Underweight]	3.67	-1.67 – 9.01	0.177
Time [9] × BMI group [Underweight]	3.61	-1.76 – 8.97	0.187
Time [10] × BMI group [Underweight]	2.56	-3.00 – 8.12	0.366
Time [11] × BMI group [Underweight]	0.51	-6.23 – 7.25	0.882
Time [12] × BMI group [Underweight]	-0.92	-7.66 – 5.83	0.789
Time [13] × BMI group [Underweight]	-0.82	-7.56 – 5.93	0.812
Time [14] × BMI group [Underweight]	0.08	-6.06 – 6.22	0.979

Time [15] × BMI group [Underweight]				1.96	-4.37 – 8.30	0.543
Time [16] × BMI group [Underweight]				0.03	-6.87 – 6.94	0.992
Time [17] × BMI group [Underweight]				3.25	-3.66 – 10.16	0.355
Time [18] × BMI group [Underweight]				2.24	-4.89 – 9.38	0.537
Time [19] × BMI group [Underweight]				-3.59	-10.86 – 3.68	0.332
Time [20] × BMI group [Underweight]				-6.28	-15.08 – 2.51	0.161
Time [21] × BMI group [Underweight]				-8.23	-17.81 – 1.36	0.092
Time [22] × BMI group [Underweight]				-5.97	-14.25 – 2.31	0.157
**Random Effects**						
σ^2	25.43			23.55		
τ_{00}	108.18 $_{Number}$			78.70 $_{Number}$		
ICC	0.81			0.77		
N	39 $_{Number}$			39 $_{Number}$		
Observations	481			481		
Marginal R^2 / Conditional R^2	0.180 / 0.844			0.405 / 0.863		
